# Polyurethane foam degradation combining ozonization and mealworm biodegradation and its exploitation

**DOI:** 10.1007/s11356-025-36029-8

**Published:** 2025-02-08

**Authors:** Margarita Ros, Paula Lidon, Angel Carrascosa, Marta Muñoz, Maria Virtudes Navarro, Jose Maria Orts, Jose Antonio Pascual

**Affiliations:** 1 Department of Soil and Water Conservation and OrganicWaste Management, Centro de Edafologia y Biología Aplicada del Segura (CEBAS-CSIC), University Campus of Espinardo, 30100 Murcia, Spain; 2 CETEM, Materials, Adhesion and Polymers, C/ Perales S/N, Yecla, Murcia, Spain; 3 Department of Biochemistry and Molecular Biology, Facultad de Farmacia, C/Prof., Universidad de Sevilla, García Gonzalez 2, 41012 Seville, Spain

**Keywords:** *T. molitor*, Frass, Chitin, Gut microbiome, 16SrRNA, Ozonization

## Abstract

The biodegradation of polyurethane foam (PU foam) using a combination of oxidative pre-treatment (ozonization) and *Tenebrio molitor* (*T. molitor*) mealworms was conducted in this study. Different degrees of ozone oxidation (0%, 25%, and 50%) were applied to PU foam, which was subsequently fed to mealworms. The mealworms’ survival and growth were then compared to mealworms receiving a normal diet (bran). Results showed that mealworms fed with non-oxidized PU foam (PUF0) exhibited a higher consumption rate (11.8%) than those fed with 25% (PUF25) and 50% (PUF50) oxidized PU foam (7.7% and 5.7%, respectively). The survival rate was similar across all the PU foam diets and the bran diet. Fourier transform infrared (FTIR) spectroscopy and differential scanning calorimetry (DSC) analyses revealed minor structural changes in the PU foam. The gut microbiota analysis showed a significant correlation between the PU foam and bran diets. Among the different oxidized PU, distinct microbial community profiles were also observed, with the genus *Klebsiella* consistently present across the PU foam diets. The ozone pre-treatment altered the palatability and degradation of the PU foam by mealworms, while the mealworm frass and chitin obtained could potentially be used as resources for agricultural and industrial applications that would close the circular bio-economy cycle.

**Supplementary Information:**

The online version contains supplementary material available at 10.1007/s11356-025-36029-8.

## Introduction

Nearly 50 million mattresses are produced in the European Union each year, and they have between 2 and 30 kg of polyurethane foam. Polyurethane foam (PU) is an open cellular structure made by reacting polyols and di-isocyanates. To produce high-quality PU foam products, the foam is used with a series of additives, depending on the application. In the European Union, 49% of mattresses are landfilled, 33% are sent to energy plants, and 17% are recycled (EBIA 2020). The Landfill Directive obliges EU Member States to reduce the fraction of municipal waste that is landfilled by 2035 to 10% or less due to environmental risk (Landfill Directive 2018/850/EC n.d). In addition, the waste-to-energy must be significantly reduced to enhance other end-of-life solutions. Research in new recycling technologies for polyurethane foam is needed to reduce its impact on the environment.

The rate at which PU biodegrades depends greatly on its material composition and properties (Bhavsar et al. 2023). Therefore, using pre-treatments that promote PU biodegradation by larvae is an alternative that has been scarcely studied. Oxidative pre-treatment of PU foam can improve mealworm biodegradation due to the loss of hydrophobicity of the isocyanate groups, increasing cell adhesion (Domansky et al. 2013; Xie et al. 2019). The oxidative attack by ozone causes the depolymerization of polyurethane, leading to gradual weight loss through the progressive destabilization and decarboxylation of the long, linear PU molecule (Patent 2024-0038 n.d; Orts et al. 2025), making it more accessible for enzymatic and microbial degradation.

Different studies have highlighted the role of some insects (yellow mealworms (*Tenebrio molitor*), wax moths (*Galleria mellonella*), and super worms (*Zophobas atratus*)) and their gut microbes in plastic degradation (polyurethane (PU), polyethylene (PE), and polyvinyl chloride (PVC)) (Lou et al. 2020; Peng et al. 2020; Orts et al. 2023). The gut microbiota of larvae has an inherent ability to mineralize polystyrene (PS) (Yang et al. 2015; 2018). In addition, yellow mealworms’ intestinal tracts can secrete emulsifying substances to facilitate plastic biodegradation (Brandon et al. 2021). The use of some insects for plastic degradation can reduce the cost of plastic waste disposal, which would be economically beneficial, not only as a solution for polymer disposal but also for producing by-products after PU biodegradation, such as frass or chitosan.

The main characteristics of frass are its high organic matter and available nutrient content (nitrogen, phosphorus, and potassium). The composition, however, depends on the insects’ diet. Furthermore, it potentially contains microbes that promote plant growth and suppress pathogens by releasing plant hormones (Poveda et al. 2019). The body of mealworms contains chitosan. Chitosan due to bacteriostatic properties and polycationic nature has many industrial, medical, pharmaceutical, and environmental applications (Pal et al. 2021; Majeti and Kumar 2000; Croisier and Jérôme 2013). In agriculture, chitosan has been proven to stimulate plant growth and yield, promote antifungal activities against numerous phytopathogenic fungi, and induce abiotic and biotic stress tolerance (Katiyar et al. 2015; Hasaneen et al. 2022; Rozman et al. 2019; Rabêlo et al. 2019).

The hypotheses of this work are as follows: (i) ozone oxidation can affect palatability for larvae, and if they ingest it, their microbiota may be different depending on the degree of ozonization. (ii) No residue of PU is observed in the frass, and it, therefore, could be used as an organic amendment. (iii) The chitosan obtained from mealworms fed with PU showed no differences from the chitosan of those fed with bran.

## Material and methods

### Mealworms and PU foam

The PU foam (polyether-PU) was purchased from Interplasp (Yecla, Murcia, Spain). This foam was cut into regular cubes (length × width × height = 2 × 2 × 2 cm) prior to the degradation experiments. Ozone generator was provided by DEPURANOVA (Camas, Spain) equipped with a redox detector (ETATRON, Italy).

For the ozonization treatment, 30 g of PU foam was placed in a reactor in water with temperature control (25 °C) and agitation (500 rpm), the gaseous ozone was injected, keeping in saturation (7–10 ppm), and its level was controlled by a redox detector (Orts et al. 2025; Patent 2024-0038 n.d). The process stopped at 24–36 h for 25% and 48–56 h for 50% foam degradation. Once each process was completed, the foam was washed three times with distilled water. Afterwards, it was dried for 24 h at 65 °C. Subsequently, foam pieces with a final weight of 0.225–0.25 g (PUF25) and 0.15–0.16 g (PUF50) were selected.

Mealworms of *T. molitor*, size ~ 2 cm (50–60 days), were purchased from BioInsecta (Molina de Segura, Murcia, Spain). The mealworms were fed with wheat bran, and the day before starting the experiment, they were starved. The experiment was run in triplicate by feeding 1 g of PU foam with different degrees of ozonization (0%, 25%, and 50%), respectively, PUF0, PUF25, and PUF 50, and 1 g of wheat bran (bran) as the control diet to 10 g (130–150 larvae) of randomly selected mealworms for 21 days. The containers were incubated in the dark at 27 ± 1 °C and 80 ± 3% relative humidity (Orts et al. 2023; Machona et al. 2022).

### *T. molitor* survival and characterization of PU foam degradation

The PU foam and mealworms were sampled at 7, 14, and 21 days to evaluate the PU foam biodegradation. The dead mealworms (appearing completely black) were counted. The mealworms, PU foam, and frass (feces) were separated and stored at 4 °C. Three mealworms were taken out and frozen for further analysis. The PU foam and the mealworms were weighed after a simple surface cleaning to calculate the mealworms’ weight loss, survival (%), and PU foam consumption (%).

The changes in the PU foam after being fed to the yellow mealworms were characterized. A control sample of PU foam (not eaten by mealworms) was also examined for comparison. Various technologies were used. Infrared spectroscopy in attenuated total reflectance (IR-ATR) mode was used for the qualitative chemical analysis. A Bruker Alpha II spectrophotometer equipped with a diamond-tipped ATR accessory was employed for this. The resulting spectra were obtained after recording 24 scans with a resolution of 4 cm^−1^ from 400 to 4000 cm^−1^. Differential scanning calorimetry (DSC) is a technique used for the thermal analyses of reversible processes with heat exchange that occur in certain materials, specifically polymeric materials. A TA Instruments Q200 calorimeter and a sample quantity of 1–4 mg were used in our study. During the analysis, the sample was subjected to a temperature sweep from − 85 to 180 °C at a heating rate of 5 °C/min in an inert nitrogen atmosphere. Scanning electron microscopy (SEM) analyzed the structural morphology of the samples. A Hitachi S-3500N scanning electron microscope was used for this purpose. The samples were previously metalized with gold, and the images were taken at different magnifications (100 and 250 ×).

### DNA extraction, amplification, and sequencing

The DNA extracted by the DNeasy PowerSoil kit (Qiagen, Hilden, Germany) from the gut of three mealworms from the same feed container was pooled to eliminate individual variability after 21 days. The mealworm gut was added to a tube with 100 µL of phosphate buffer (0.1 M). The quantity and quality of the DNA extracts were evaluated using a Qubit 3.0 Fluorometer (Invitrogen, Thermo Fisher Scientific, Waltham, MA, USA). The hypervariable region V3–V4 of the bacterial 16S rRNA gene was amplified using the primer pairs 341F (5′-CCTACGGGNGGCWGCAG-3′) and 785R (5′ GACTACHVGGGTATCTAATCC-3′). Purified amplicons were pooled in equimolar amounts and paired-end sequenced on an Illumina MiSeq v3 600 (Illumina, San Diego, USA), following the standard protocols established by the Unidad Genomica IPBL, Instituto De Parasitologia y Biomedicina “LÓPEZ-NEYRA.”

### Bioinformatic analysis

The demultiplexed fastq file quality was tested using the FASTQC program v0.12.1 (Andrews 2010) and processed using the DADA2 v1.22 pipeline (Callahan et al. 2016) on R v4.1.2 (R Development Core Team 2021) from Rocky Linux. We trimmed the primers at 19 and 21 nucleotides and truncated the sequences at the first nucleotide with a quality score of three. We merged the forward and reverse sequences with a minimum overlap of 12 and removed the chimeras using the “consensus” method. We used the SILVA v138.1 database (Quast et al. 2013) to assign the bacterial taxonomy. The ASVs not assigned to a known phylum were removed. The ASVs were rarefied to the lowest sequencing depth found (20,370 reads) belonging to 203 ASVs. We used the FAPROTAX database (Louca et al. 2016) to estimate the potential functionality of the bacterial community.

### Characterization of frass

The frass was analyzed to ensure that no PU foam was left. The analyses used were IR-ATR, DSC, TGA, and SEM, following the protocol in the “T. molitor survival and characterization of PU foam degradation” section. In addition, the human pathogens *Salmonella* and *E. coli* were analyzed according to the proceeding PNT-04 (qPCR) by Microgaia Biotech (Murcia, Spain).

### Chitin extraction and characterization

The extracted chitin from *T. molitor* was analyzed using the following protocol:

1. Drying and grinding the Tenebrio sample.
2. Demineralization: 1 M HCl was used. After 50 min at 80 °C, the resulting sample was washed and filtered.
3. Deproteinization: 1 M NaOH was used at 80 °C for 18 to 24 h (again, the samples were washed and filtered).
4. Bleaching: carried out with a ratio of 5 g of dry content per 100 ml of H_2_O.

To analyze the structure of the chitin, a Fourier transform infrared spectroscopy (FTIR) test was conducted using the Bruker Tensor-27 spectrophotometer. Elemental analysis was performed using the FlashSmart™ Elemental Analyzer (ThermoFisher Scientific™, MA, USA) to determine the nitrogen, carbon, and hydrogen content in the chitin extracted from *T. molitor*. The degree of acetylation (DA) was calculated from the C/N ratio using the following formula:

$$DA=((C/N-5.14)/1.72)\times 100$$

### Statistical analysis

R v4.1.2 (R Development Core Team 2021) for Windows was used for statistical analysis. A one-way ANOVA followed by a post hoc Tukey’s HSD test was performed to determine the differences among the ozone concentrations in the PU consumption. We used the microeco (Liu et al. 2021) package to calculate the alpha diversity indexes through the *cal_alphadiv* function from microeco. The community’s structure was plotted by conducting a principal coordinate analysis (PCoA) based on the Bray–Curtis dissimilarity matrix, calculated using the *cal_betadiv* function from microeco. To determine the differences among the treatments, a one-way PERMANOVA with 999 permutations, using the *adonis* function in the vegan package, was employed (Oksanen et al. 2022). Spearman’s correlation coefficients between the bacterial taxa and the percentage of PU consumed were calculated using the *cal_cor* function with *fdr* correction from microeco. A Venn’s diagram was made using the *trans_venn* function from microeco. We studied the changes in the abundance of 12 bacterial genera of the PUF0, PUF25, and PUF50 ozonization treatments compared to the control, calculating the natural log response ratios (LRR) and their confidence intervals at a 95% confidence level using a modified version of the function *logRespRatio* from the ARPobservation package (Pustejovsky 2023). We conducted a Kruskal–Wallis rank sum test to determine whether the relative abundances of bacterial families and genera differed among the treatments.

## Results and discussion

### Oxidative treatment of PU foam

An oxidative pre-treatment was applied to the PU foam to increase its susceptibility to biological attack by destabilizing and breaking the polyurethane molecule, modifying its crystalline structure, and reducing its hydrophobicity and therefore its resistance to biological degradation (Xie et al. 2019). The action mechanism of the oxidative attack performed on the PU foam explains that this treatment destabilizes the ester bond of the isocyanate, followed by progressive decarboxylation of the long and linear PU molecule (Orts et al. 2025). This process reduces the crystalline structure and, thus, the rigidity and hydrophobicity of the foam. According to our hypothesis, this would promote greater foam consumption by the *Tenebrio molitor* larvae, with its decomposition completed through the synergistic action of chewing and the enzymatic activity in the worms’ digestive tract.

### PU foam consumption and mealworm survival

**Fig. 1:**
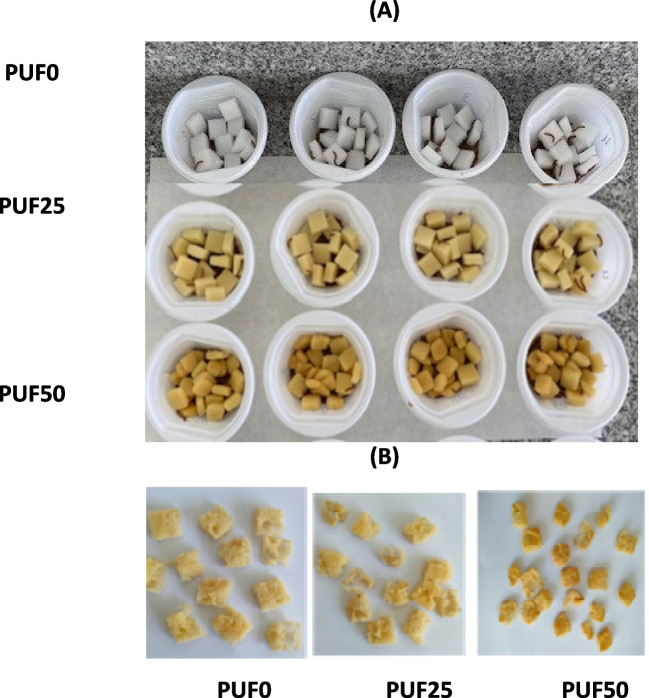
PU foam eating behavior of mealworms (*T. molitor*) at 0 days (**A**) and after 21 days (**B**)

The mealworms chewed the PU foam, as we observed how they dug into the foam structure (Fig. 1B). The mealworms’ eating activity was higher in feed at PUF0 than in feed at PUF25 and PUF50 (Fig. 1A, B).

**Fig. 2:**
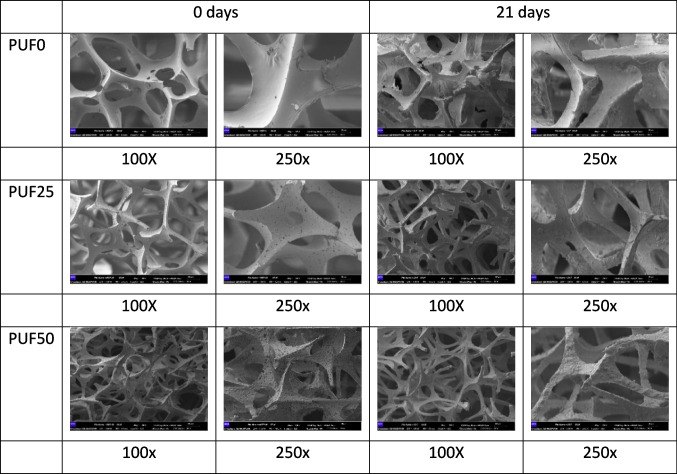
SEM observations of the physical surface topography of the three PU foam samples (PUF0, PUF25, and PUF50) not eaten by mealworms (0 days) and eaten by *T. molitor* after 21 days (100 × , 250 × magnification)

The smooth fiber surface, from the structure of the pristine PUF0, was intact (Fig. 1A). After 21 days, the structure was fractured and with remains that could have come from contact with the mealworms: wax or frass residues (Fig. 2). This may explain the yellowing of the PUF0 after the mealworms ate it. The PUF25 and PUF50 ozonization prior to exposure to the mealworms showed pores in the polymer ribs that increased with the degree of ozonization (Fig. 2). After using this foam as feed for *T. Molitor*, no major deterioration of the foam structure was observed. However, there were remains on the surface of the PUF from the feeding process of mealworms.

The survival of the mealworms with the control diet was 99%, while the survival of the mealworms with PUF0, PUF25, and PUF50 was 96%. The survival rate with the different treatments could indicate that PU foam feeding had no negative impact on the survival capabilities of the mealworms, regardless of the ozonization percentage of the PU foam. This is contrary to the findings by Liu et al. (2022), where the survival rate was lower. The different characteristics of the PU used in the studies could explain these results. The weight of the mealworms fed with PU foam ranged from 8.0 to 8.9 g, corresponding to a 10–20% weight loss throughout the experiment. The mealworms fed with bran did not show any weight loss, ranging in weight from 9.8 to 10 g. This could be due to bran containing nutrients for the *T. molitor* mealworms’ metabolism (Peng et al. 2019), while PU foam as the only source of carbon would not provide sufficient nutrients to support growth. Orts et al. (2023) observed similar results.

**Fig. 3:**
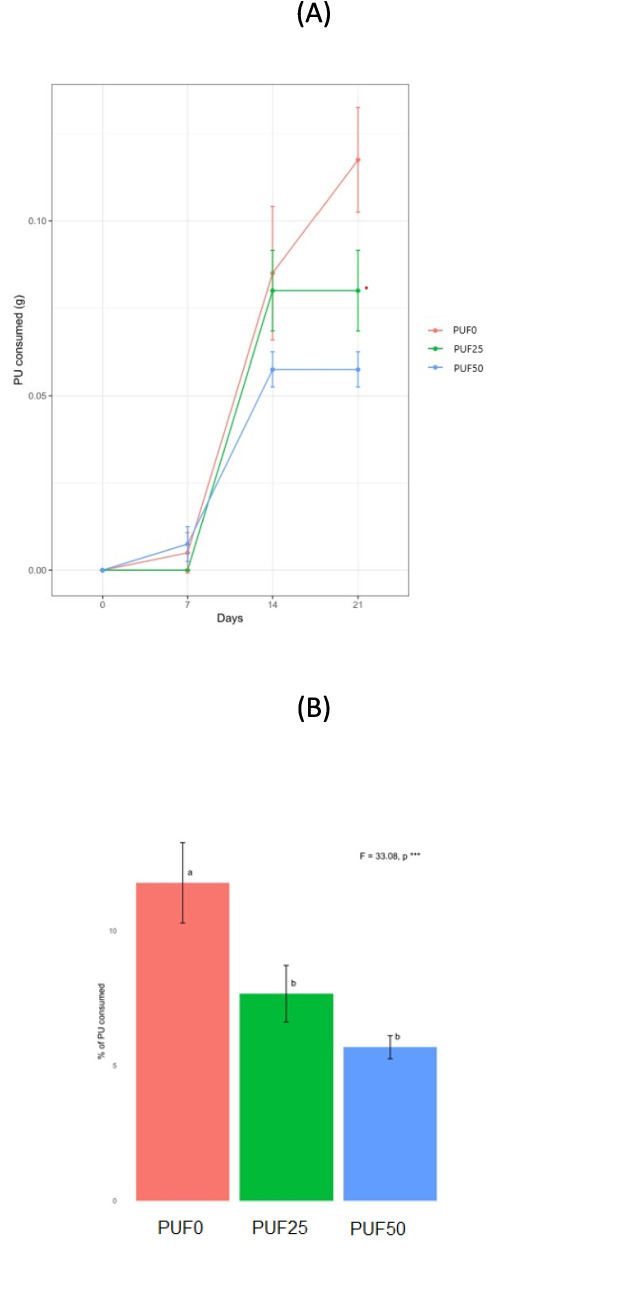
Accumulative PU consumption (g) (**A**) and percentage total PU foam consumption (**B**). Different letters correspond to significantly different groups according to Tukey’s HSD test

The consumption of PU foam increased during the experiment (Fig. 3A). The mealworms fed with PUF0 consumed more (11.8%) than the ones fed with PUF25 and PUF50 (7.7% and 5.7%, respectively) (Fig. 3B). The mealworms had to adapt to PU foam feeding as indicated by a slower consumption rate during the first 7 days. After adaptation, the PU foam consumption rate increased during the following 7 days and then slowed down later, implying that the upper limit of PU foam consumption was approaching (Fig. 3B). The reaction of polymers to ozone causes the chain to split and oxidize, constantly degrading its mechanical properties (Xie et al. 2019). It could be possible that PU oxidization generates some secondary products or a more adhesive surface that is not appetizing for mealworms.

The average PU foam consumption was approximately 0.049 mg day^−1^ per worm in the PUF0, but it decreased to 0.019 mg day^−1^ per worm in the PUF25 and PUF50. These are lower values than those observed in previous studies with other polymers, e.g., polyethylene (PE), polypropylene (PP), and polystyrene (PS) (Brandon et al. 2018; Yang et al. 2021a, 2021b), and with PU (0.18 mg day^−1^ per worm) (Liu et al. 2022). This could be due to the types of polyols employed (Liu et al. 2022), the number of PU layers in the foam, or even the age or origin of the mealworms. Yang et al. (2018) pointed out that when using *T. Molitor* to degrade different plastics, the density of the plastic had a significant effect on the degree of biodegradation. This could be why the more oxidized, denser PUF50 was more difficult for the worms to eat. One important consideration is that the origin and specific characteristics of PU foam, such as crystallinity and surface texture, can significantly influence the biodegradation rate (Stefaniak and Masek 2021).

### Biodegradation of PU foam

**Fig. 4:**
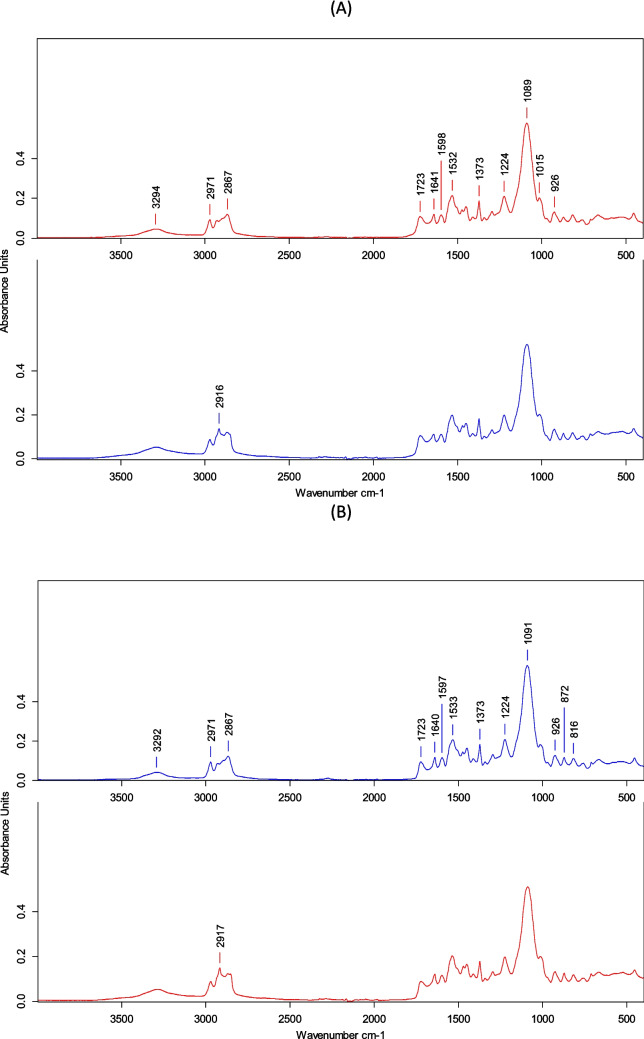

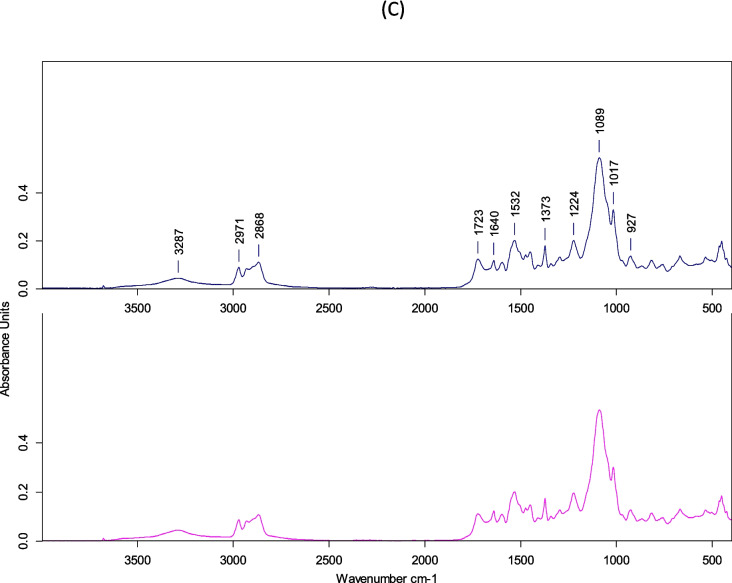
FTIR spectra of PU foam before (top) and after (bottom) mealworm feeding on the three different ozonized PUF0 (**A**), PUF25 (**B**), and PUF50 (**C**)

FTIR spectroscopy was used to analyze the modified functional groups of the different PU foam samples and compare them to the pristine ones after 21 days of mealworm biodegradation (Fig. 4). The PU foam showed peaks characteristic of polyurethane (Wang et al. 2022). The residual PUF0 showed lower peak intensity than the pristine PUF0. Samples showed a N–H stretching at 3294 cm^−1^; asymmetric and symmetric C–H bond stretching of the methyl and methylene groups (2971 and 2868 cm^−1^)); carbonyl group, –C = O–, stretching of urethane (1722 cm^−1^); –C = O– bond stretching of the urea group (1641 cm^−1^), C–C aromatic stretching bond (1598 cm^−1^); –C–N– stretching in the urethane group (1533 cm^−1^); and asymmetric and symmetric –C–O– bond stretching in the ether groups, –C–O–C (1224 and 1091 cm^−1^). Orts et al. (2023) showed similar values, considering that the PU molecule was occluded and only the external parts could be degraded by microorganisms through extracellular enzymes (Wang et al. 2022), without observing modifications in the hard segments (such as the breaking of the free aromatic bond (Christenson et al. 2006) or in the urethane ester/ether bond in the soft segments, such as the plane urethane represented by C–N of N–H (Wang et al. 2022)). In contrast, Liu et al. (2022) and Zhang et al. (2012) observed some disappearances of –C = C– (benzene rings 1534 cm^−1^) and –C–O–C–, indicating the scission of the ether bond in the soft fragment of PU foam and the disappearance of –C = O– in residual PU foam, implying the hydrolysis of the urethane bond in PU foam. We also observed a C–H stretching vibration (2916 cm^−1^) in the residual PU foam that is typical of wax or even guano residues, which remain as residue on the surface of the foam after being in contact with the *Tenebrio Molitor* during the feeding process. This could explain the coloration differences between the residual PUF0 and the pristine PUF0.

**Fig. 5:**
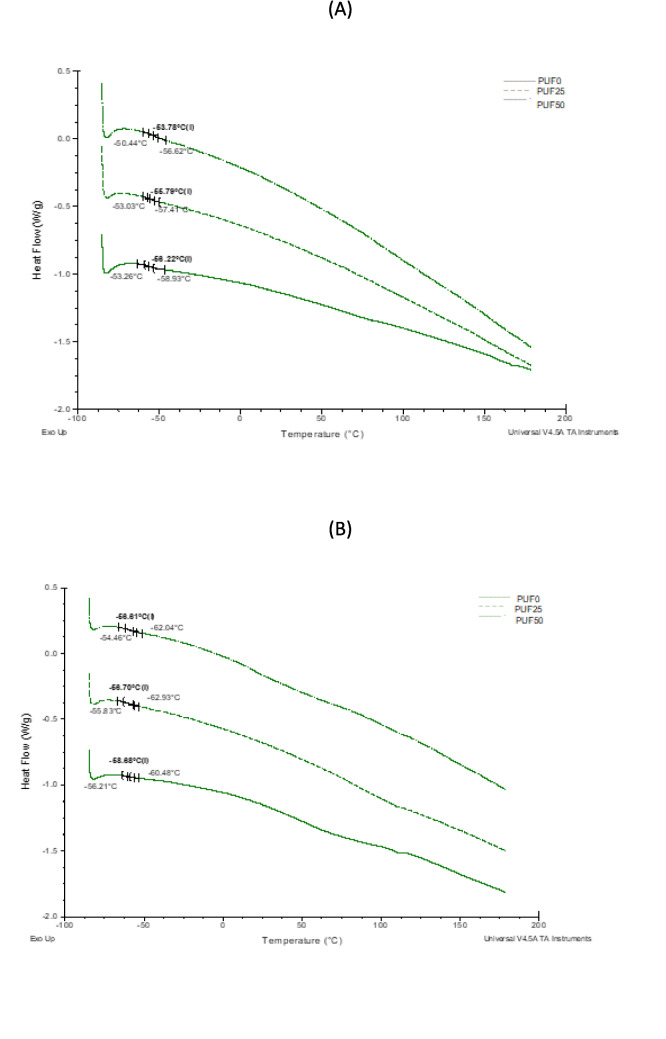
DSC curves of PU foam at different ozonization degrees (**A**) and residual foam after feeding mealworms for 21 days (**B**)

DSC is a thermal analysis technique useful for determining the glass transition temperature (Tg) of polymeric materials. This is related to the material’s structure and, specifically, to the internal volume of the polymeric material. The results obtained from the DSC analysis of the pristine PU foam and PU foam after ozonization (Fig. 5A) show a slight increase in the Tg value with increased degrees of ozonization. This observation indicates that the degree of ozonization produces structural changes in the foamed materials, leading to greater difficulty in moving the chains that make up the amorphous part of the polyurethane. However, when the same formulation is used to feed mealworms, a slight decrease in the Tg value is observed (Fig. 5B), which is more notable in the non-ozonized PUF0. This decrease in the Tg value of the foam after being fed to mealworms indicates easier movement of the polymer chains in the amorphous region, which may be related to structural changes in the polymeric material (Table 1).

**Table 1:** Tg values of PU foam after different ozonization degrees before and after feeding mealworms for 21 days

Ozonization degree	Before feeding mealworms	After feeding mealworms
0%	− 56 ± 1 °C	− 59 ± 1 °C
25%	− 56 ± 1 °C	− 57 ± 1 °C
50%	− 54 ± 1 °C	− 57 ± 1 °C

### Gut microbial communities after PU foam consumption

**Fig. 6:**
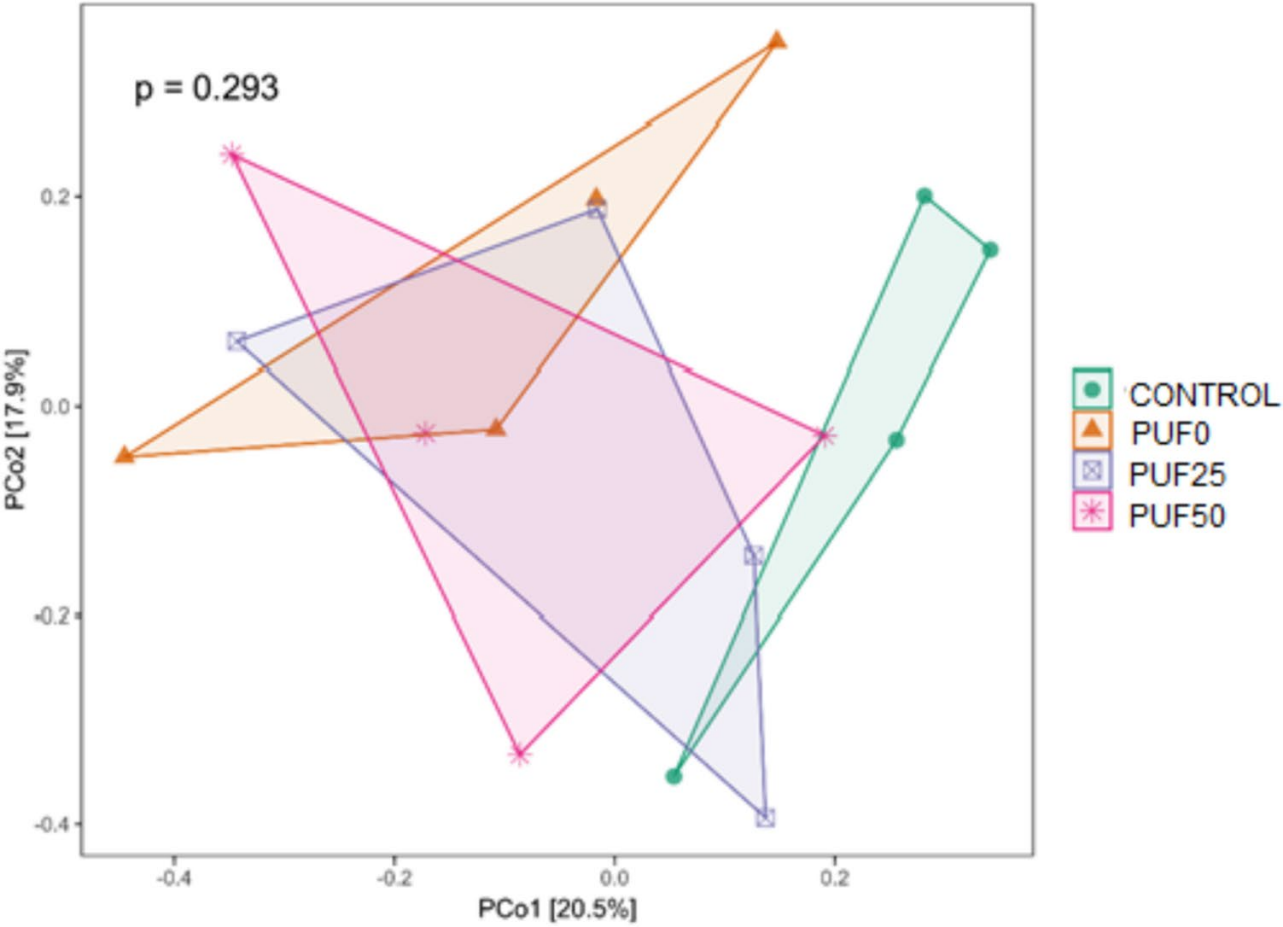
Principal coordinate analysis (PCoA) showing the Bray–Curtis dissimilarity matrix of the gut bacterial structure after different feeds

The analysis of diversity within a single sample measured by Shannon, Simpson, and observed ASVs showed no significant differences between the different feeds (Fig. S1). In addition, a principal component analysis (PCoA) based on the Bray–Curtis dissimilarity matrix revealed that the control diet gut microbiota (fed with bran) was grouped in one cluster. While, the other PU foam diets (PUF0, PUF25, and PUF50) was grouped in another cluster (although the differences were not significant) (Fig. 6), suggesting that the gut microbiota shifts were associated with the PU foam, regardless of its ozonization. Orts et al. (2023) also observed different mealworm gut bacterial communities with PU foam and bran feed.

**Fig. 7:**
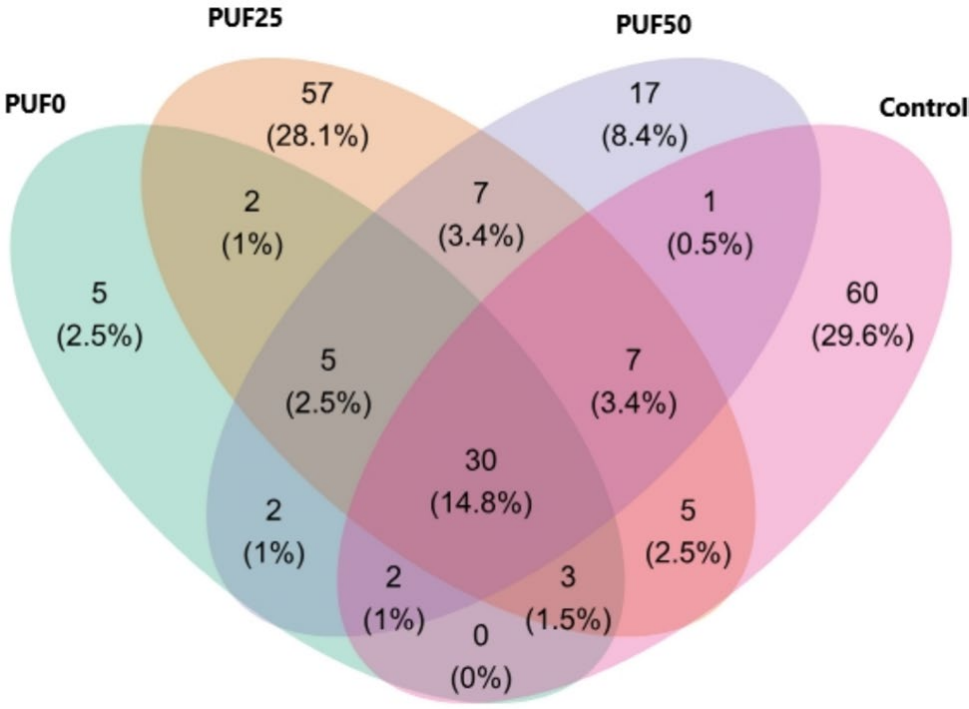
Venn’s diagram showing the number and relative percentage of the total community of unique and shared ASVs among the different feeds

The Venn’s diagram shows that 29.6% of the ASVs belong only to the control sample, while the PUF0, PUF25, and PUF50 samples showed 2.5%, 28%, and 8.4% of ASVs, respectively. The common ASVs in all the PU foam (PUF0, PUF25, and PUF50) were low (2.5%), and the ASVs were also low (3.4%) between the PUF25 and PUF50 samples. This could indicate that the percentage of ozonization significantly affected the number of ASVs in the gut microbial community (Fig. 7). The ASVs from PU foam and control samples had in common, which were low. The most significant difference between them was 2.5% (Fig. 7).

**Fig. 8:**
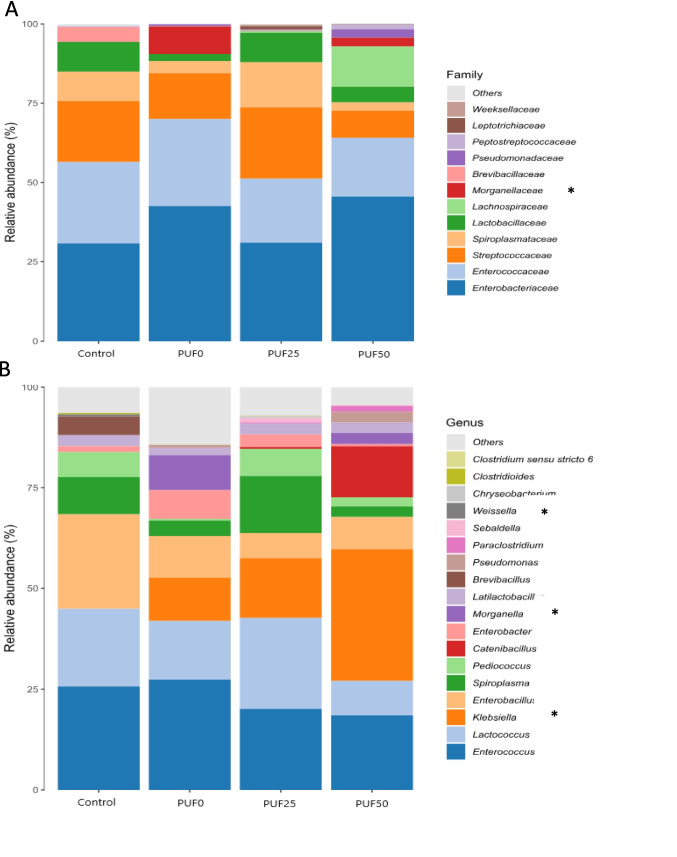
Relative abundance of family (**A**) and genus (**B**) gut bacterial community of different feeds. *Indicates significant differences among treatments (*p* < 0.05) according to the Kruskal–Wallis test

Montalbán et al. (2022) showed that diet affected the gut microbiota profile of *T. molitor*. The gut microbiome in the mealworms contained six phyla (Bacillota, Pseudomonadota, Fusobacteriota, Bacteroidota, and Actinomycetota), with no significant differences between the different feeds. In contrast to Orts et al. (2023), who showed other phyla in their mealworms fed with PU and Bran (Proteobaterias, Bacteroidetes, Firmicutes, and Tenericutes), it could be that the type of meal and, especially, the mealworms can also affect the gut microbial community. The 12 most common families found in our study were *Enterobacteriaceae*, *Enterococcaceae*, *Lactobacillaceae*, *Spiroplasmataceae*, and *Streptococcaceae*, which change according to the family and feeds. However, only the family *Morganellaceae* was significantly different among the treatments (Fig. 8). *Spiroplasmataceae*, *Enterococcaceae*, and *Enterobacteriacea* are common insect gut-associated bacteria found in the *T. molitor* microbiome (Liu et al. 2021; Yang et al. 2021b; Osimani et al. 2018). *Enterobacteriaceae*, *Enterococcaceae*, and *Streptococcaceae* showed higher abundance than *Spiroplasmataceae* and *Lactobacillaceae*. *Enterobacteriaceae* slightly increased in PU foam compared to the control (Fig. 8A). Liu et al. (2022) and Yang et al. (2014) suggested that Enterobacteriaceae (*Enterobacillus, Enterobacter*) can contribute to polyether-PU degradation and PE degrading.

**Fig. 9:**
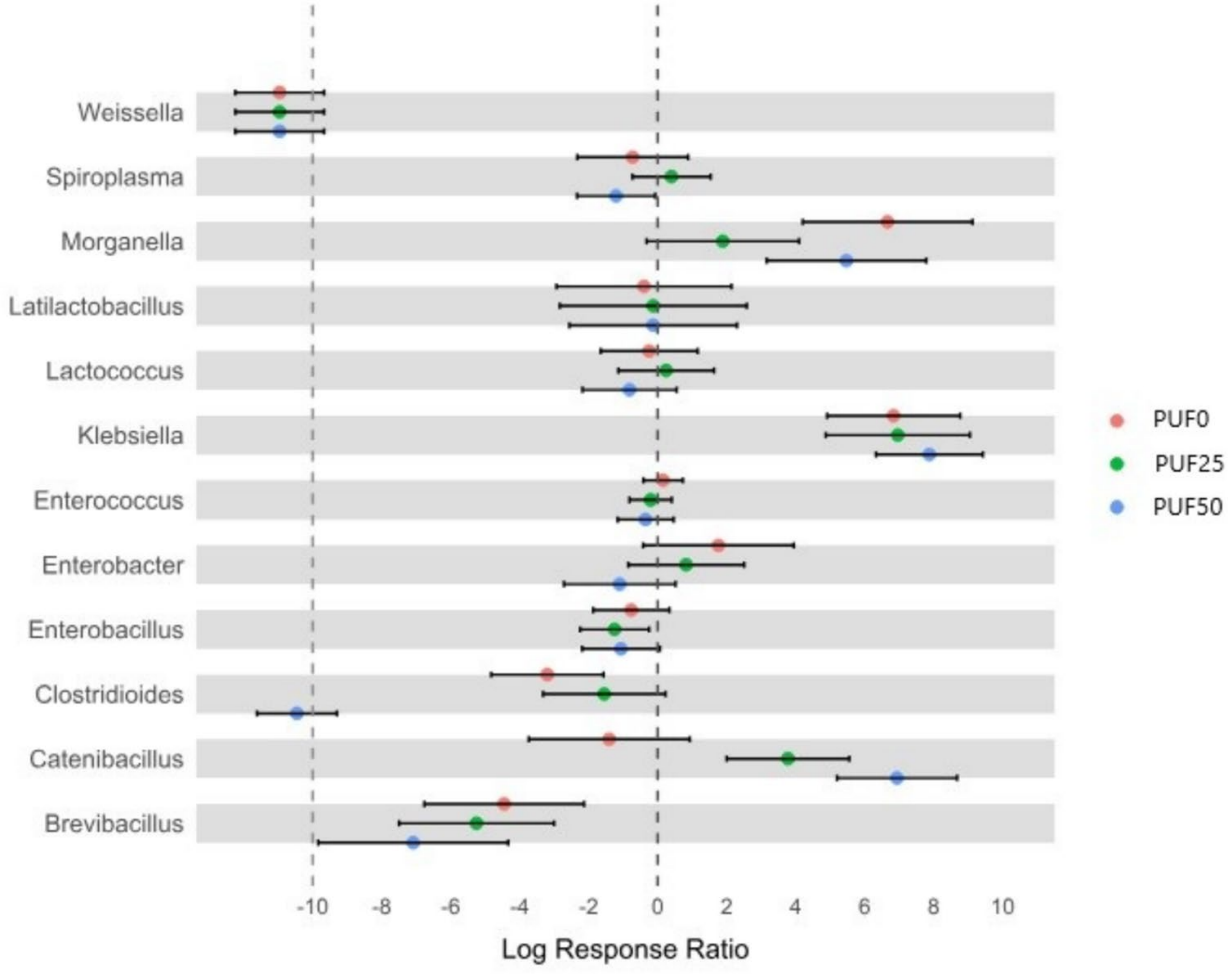
Mean log ratio response (LRR) of the genera associated with the different feeds. Values under − 10 LRR correspond to the disappearance of the genus in that treatment (relative abundance = 0)

Different abundances of 18 bacterial genera were associated with the feed: *Enterobacillus, Enterobacter, Klebsiella,* and *Morganella* (Enterobactericeae); *Enterococcus* (Enterococacciae); *Lactococcus* (Streptococcaceae); *Latilactobacillus, Pediococcus,* and *Weissella* (Lactobacillaceae); *Spiroplasma* (Spiroplasmataceae); and *Catenibascillus* and *Brevibacillus* (Paenibacillaceae) (Fig. 8B, Fig. 9). Among them, *Weissella*, *Morganella*, and *Klebsiella* showed significant differences among the treatments. *Klebsiella*, *Morganella*, *Catenibacillus*, *Spiroplasma*, and *Enterobacter* generally increased with the PU foam (Fig. 8B, Fig. 9).

*Morganella*, *Latilactobacillus*, and *Lactococcus* were more highly correlated with the PUF0; *Brevibacillus*, *Klebsiella,* and *Enterobacter* were higher for the PUF25; and *Klebsiella* and *Catenibacillus* were higher for the PUF50 (Fig. S2). These latter two are known for their involvement in the degradation of carbonyl groups and C–C bonds (Saygin and Baysal 2021; Braune and Blaut 2018). *Spiroplasma* has also been observed in many mealworms gut fed with different types of plastics (Urbanek et al. 2020; Brandon et al. 2018). It is considered a pathogen or a male-killing bacterium and it is not harmful in the mealworm gut (Jung et al. 2014). Liu et al. (2022) showed an association of *Enterobacter* with PU degradation, and Yang et al. (2014; 2021a) observed this genus associated with PP feed and LDPE. Orts et al. (2023) associated *Lactococcus* with PU-degrading. They are considered common bacteria that help maintain a stable gut microbiome and prevent the colonization of harmful bacteria (Fang 2011). *Brevibacillus borstelensis* is also associated with the degradation of the CH_2_ backbone of non-irradiated polyethylene (Hadad et al. 2005). *Morganella morganii* has also been observed bound to a plastic substrate in surface water (Ferheen et al. 2023).

*Klebsiella* is the only genus that correlated with all the types of oxidated PU foam, but the other genera are also cited in the literature as possible degraders of PU foam and other polymers. According to Urbanek et al. (2020), the digestion process in the mealworm gut is very complex and the role of whole microbiota and their synergic interactions is important in PU degradation.

### Taking advantage of mealworms

After the mealworms ingest the PU foam, providing a potential solution for PU degradation, two more mealworm components can use the frass as organic fertilizer and the mealworm’s body as chitin.

The frass obtained from *T. molitor* mealworms is used as organic fertilizer due to its rich nutrient content (Houben et al. 2020). Therefore, the frass from mealworms fed with PU foam could also be used as organic fertilizer as long as there are no demonstrated harmful effects on human health and the environment. We demonstrated that neither of the frasses showed *E. coli* nor *Salmonella*. After analysis by IR-ATR, DSC, TGA, and SEM (Fig. S3), no traces of PU foam were observed. Therefore, frass can be used as fertilizer, although this has not been widely explored.

The *T. molitor* mealworms can also be used to obtain chitin. Therefore, we tested whether any differences appeared when feeding mealworms with PU foam. After chitin extraction, no significant differences were observed in the chitin from mealworms fed with PUF0 and those fed with bran. The FTIR results (Fig. S4) show that no differences in peaks were observed, although slight differences in intensity were noted (Fig. S4). The results of an elemental analysis reveal notable differences in chitin composition depending on the type of feed (PU or bran). These differences potentially affect the degree of acetylation (Table S1), being slightly higher in the chitin from PU foam than in the chitin from bran.

## Conclusions

*T. molitor* mealworms eat PU foam, and contrary to our hypothesis, the pre-treatment ozonization negatively affected PU consumption, decreasing from 11.8 to 5.7%. The survival of the mealworms in contact with the PU foam was similar to that of those fed with bran, but the weight of the mealworms decreased due to the lack of nutrients from the PU foam. The PU foam showed no significant changes after mealworm feeding, as observed by FTIR and TGA.

The gut microbiota of the mealworms fed PU foam differed from that fed bran. Distinct genera are associated with different percentages of ozonization, demonstrating the relationship between PU foam and the gut microbiome. Moreover, the frass and chitin from mealworms fed with PU foam, in addition to bran-fed mealworms, can be used for agriculture or industry. In this way, we could use mealworms to degrade PU foam and avoid PU foam-related environmental problems, as well as exploit other resources from mealworms as part of the circular economy**.**

## Supplementary Information

Below is the link to the electronic supplementary material.

Supplementary file1 (DOCX 678 KB)

## Data Availability

Data is available under the requirements.
