# Supplementary material for: Polyurethane foam degradation combining ozonization and mealworm biodegradation and its exploitation
Source: Environ Sci Pollut Res Int. 2025 Feb 8;32(9):5332–46. doi: 10.1007/s11356-025-36029-8 (PMC11868246; doi:10.1007/s11356-025-36029-8)
Supplement: Supplementary file 1 — Supplementary file1 (DOCX 678 KB) [file 11356_2025_36029_MOESM1_ESM.docx]

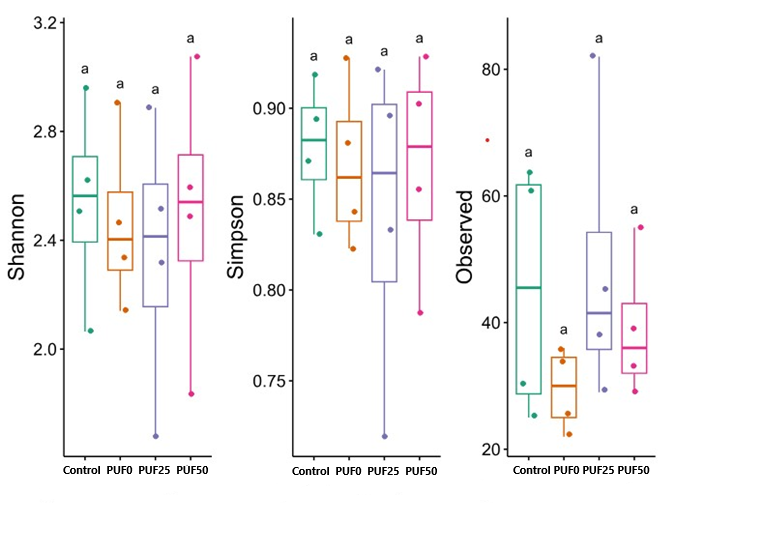


**Fig. S1.** Alpha diversity index of microbial gut of different feeds


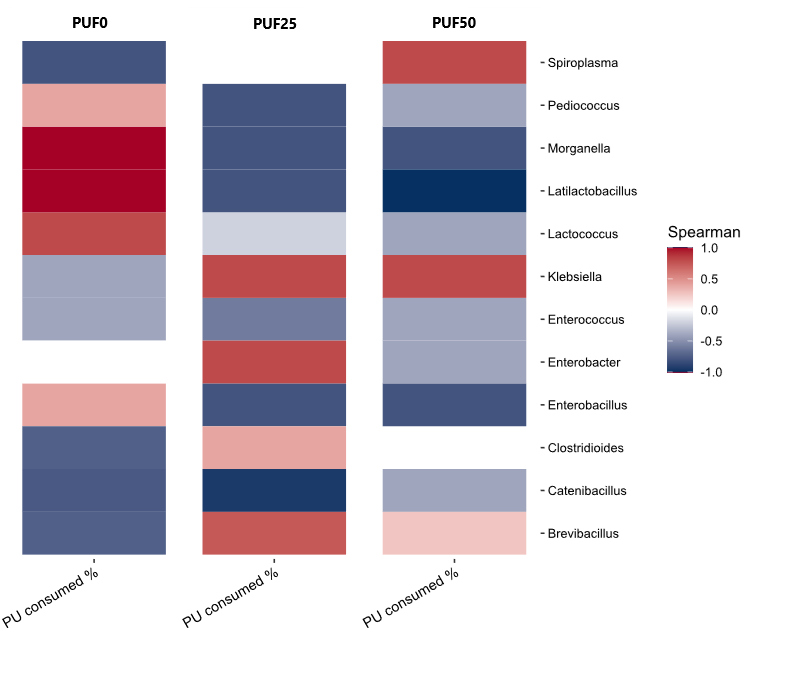


**Fig. S2** Spearman correlation of different genera with the total % of PU consumed for each type of PU foam.

| Frass from mealworms fed with bran | | |
| --- | --- | --- |
|  |  |  |
| 100x | 250x | 1500x |
| Frass from mealworms fed with PU foam | | |
|  |  |  |
| 100x | 250x | 1500x |

**Fig. S3.** SEM images of frass from mealworms fed with bran and fed with PU foam

**
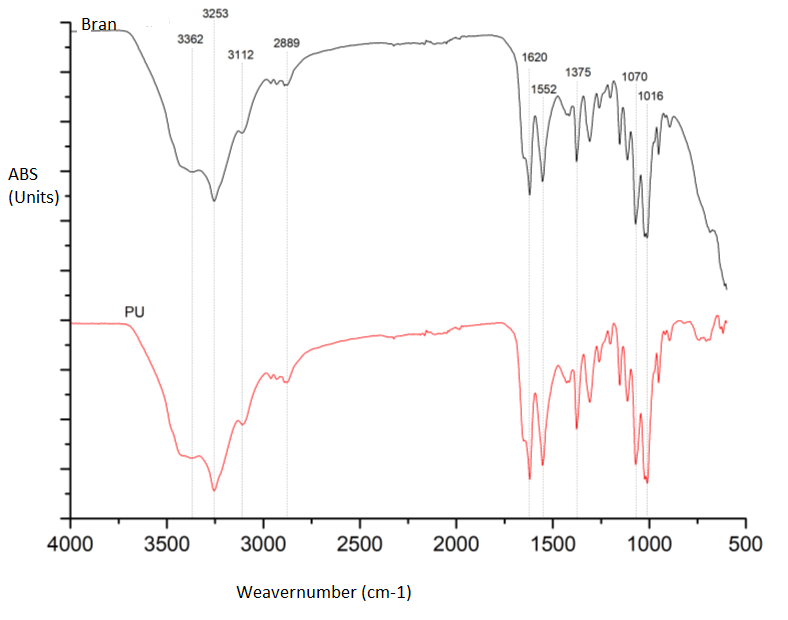
**

**Fig. S4.** FTIR spectra of chitin from bran (black line) and PU (red line)

**Table S1.** The degree of acetylation calculated from the elemental analysis.

| **Chitin** | **%N** | **%C** | **%DA** |
| --- | --- | --- | --- |
| Bran | 2.25 ± 0.05 | 15.55 ± 0.45 | 104 ± 2.7 |
| PU foam | 1.91 ± 0.08 | 13.48 ± 0.41 | 115.5 ± 1.4 |
